# Comparison of Repair Methods for Cracked Titanium Alloy Aircraft Structures with Single-Sided Adhesively Bonded Composite Patches

**DOI:** 10.3390/ma16196361

**Published:** 2023-09-22

**Authors:** Junshan Hu, Chengyu Li, Jinrong Fang, Shizhan Chen, Shanyong Xuan, Wei Tian

**Affiliations:** 1 College of Mechanical and Electrical Engineering, Nanjing University of Aeronautics and Astronautics, Nanjing 210016, China; 2 State-operated Wuhu Machinery Factory, Wuhu 241007, China; 3 AVIC China Airborne Missile Academy, Luoyang 471009, China

**Keywords:** composite patch, adhesively bonded repair, interfacial microstructure, repair efficiency, processing stability

## Abstract

Composite patches are widely accepted as a useful practice for the repair of cracked aircraft components and the repair method is of vital importance to the final performance of the repaired structures. The present research experimentally studied the repair efficiency and processing stability of pre-cured, prepreg (including unidirectional and plain weave prepregs) and wet-layup methods for use on cracked Ti-alloy panels through the configuration of a butt joint bonded with a one-sided composite patch. The efficiency and stability of these repair methods were elaborately evaluated and compared via the load bearing behavior, the microstructure of the bonding interface, and the structural failure morphology through two batches of testing specimens. Typical patterns were found in load-displacement curves where the initial damage and ultimate bearing load points divided them into elastic-linear, damage propagation and complete fracture phases. Although the co-cure process of both unidirectional prepreg and wet-layup methods can form a jigsaw-like demarcation interface between the adhesive layer and the composite patch to achieve a good bonding force and a high recovery of loading performance, the latter presents porous patches with a high coefficient of variation in load-carrying capacity. Conversely, the pre-cured laminate and the plain weave prepreg patches failed to restore the mechanical properties owing to the weak bonding interface and the low axial patch strength, respectively. The unidirectional prepreg patch was proven to be the optimal repair method for the cracked metallic structures when balancing repair efficiency and processing stability.

## 1. Introduction

The titanium alloy (Ti-alloy) finds wide applications in the fields of aerospace, defense and automobiles owing to its superior features including its low density, robust specific strength, and corrosion resistance [1]. The usage of Ti-alloy components in high-speed airplanes can lighten the structural weight, improve the loading quantity, and enhance maneuverability [2]. However, as with other metallic materials, Ti-alloy airframes are prone to cracking under the extreme loading spectrum during their years of service life [3]. The crack damage in Ti-alloy structures is a critical problem to be solved for the structural integrity and performance recovery in the maintenance of aged aircrafts [4]. The adhesively bonded repair of cracked Ti-alloy structures with a composite patch, an important means of repair, has many advantages over traditional methods such as minimal additional weight, alleviated stress concentration around cracks, and efficient load transfer without inducing new stress defects [5]. Despite its merits, the composite patch repair on cracked Ti-alloy components is usually a nonconventional bonding process since the cracks with a nonuniform location and size occur randomly in airframe structures. Therefore, targeted investigation is necessary to seek composite patch repair methods with a good repair efficiency, stable processing stability, and manual implementation convenience.

The repair of metallic airframe components based on bonded composite patches began in the 1970s, with Baker the pioneer in this field [6]. Following this, many scholars conducted numerous studies on the repair ingredients in the repair system. In terms of patch materials, glass–epoxy, carbon–epoxy, boron–epoxy, graphite–epoxy, aramid–epoxy as well as their hybrid patches were employed for the repair of cracked aluminum alloy (Al-alloy) components [7,8,9,10,11]. Among these, carbon fiber composites exhibited a higher elastic modulus and tensile strength, while the incorporation of aramid fiber layers effectively improved the bending behaviors of carbon fiber patches. Different from fiber reinforcements, the epoxy resin type was found to have a very limited influence on the repair effectiveness [12]. Structural adhesives were utilized as a bridge between patches and cracked adherends to restore structural integrity and form a load transfer path [13]. Although the ductile adhesive was used in the single-lap bonded joints to alleviate stresses more evenly along the bonded area [14], the stiff adhesive was adopted in the repair to help close the crack and to withstand higher external loads [15]. Particularly, the arrangement of functionally graded bondlines in lap joints with the same brittle adhesive for the central part, yet different ductile parts for the tip region of the overlap could enhance the joint strength [16]. The carbon–epoxy composite patches together with a stiff structural adhesive were the most common combination and were therefore used for the present research.

As well as the repair materials, the patch configuration and repair process were also the research focus [17]. Different patch shapes including forms of circle, triangle, rectangle, trapezoid, ellipse, and octagon were applied to center-cracked or V-notched Al-alloy plates [18,19,20]. It was found that the octagonal patch performed best in alleviation of stress concentration, while the rectangular shape provided the most efficient loading capability. Additionally, the composite patch layup was reported to affect the structural stiffness, loading strength, and damage progression; a layup towards 0° adjacent to the adhesive interface was highly recommended [21,22]. For the repair process, composite patches were tailored from pre-cured laminates [23] or prepared with wet prepregs [24]. The pre-cured patch was more common and convenient for performing remediation, whereas the co-cure of the prepreg patch achieved better conformability to the repaired components. Through the repair tests on honeycomb sandwich structures, the latter showed much higher bondline strengths and better structural stability than the former [25]. Moreover, the application of a pre-stressing force to the composite patch can produce artificial crack closure although an extra tension device was inevitable and not applicable in the outfield condition [26,27]. Another essential factor during the repair process was the curing method which included a heat compensation instrument [28], microwave [10], autoclave [29], thermos-press [30], etc., among which the first was often used in outfield repair, and the last two provided precise temperature and pressure for the curing process to acquire good forming consistency in the research.

The main goal of the patch repair was to restore structural integrity and load-bearing performance to cracked metallic components, thus the assessment of the maintenance quality was of vital importance [31]. Commonly, the repair effectiveness can be evaluated quantitatively via structural stiffness, bearing strength, fatigue, and damage tolerance in experimental investigations [32]. Moreover, the stress intensity factor (SIF) at the tip of the repaired crack was used as an index to characterize the stress reduction in repaired structures in analytical or finite element models [33,34]; it was revealed that SIF did not depend on the crack if the crack grew up below the repair. In addition, the microstructure and morphology were also good clues and were employed to qualitatively deduce the damage evolution in composite patch-repaired metallic structures [35,36].

These remarkable works contribute a lot to the repair of damaged metallic aircraft structures. However, the previous studies are mostly focused on Al-alloy components. Ti-alloy structures are also common in aircraft although the repair of them is rarely reported. Additionally, the bonding and failure mechanism must be thoroughly clarified in order to support the mechanical behaviors as well as to further process optimization. Moreover, the processing stability and implementing conveniences of composite patch repair in the outfield condition deserves more attention except for the common repair performance in a laboratory environment.

In this research, the pre-cured, prepreg and wet-layup composite patch methods were applied to the repair of a completely cracked Ti-alloy plate configuration. The microstructure of the bonding interfaces and the static tensile properties including structural stiffness, load bearing behavior and, failure modes were utilized to evaluate the mechanical performance of repaired structures with different composite patches. The most suitable repair method for randomly occurring crack damage in the outfield condition was ascertained through comparing their repair efficiency and processing stability. It is hoped that the findings reported here will help to provide useful guidance for the manual repair of metallic aircraft structures.

## 2. Experimental Approach

### 2.1. Materials and Specimen Configurations

**Figure 1:**
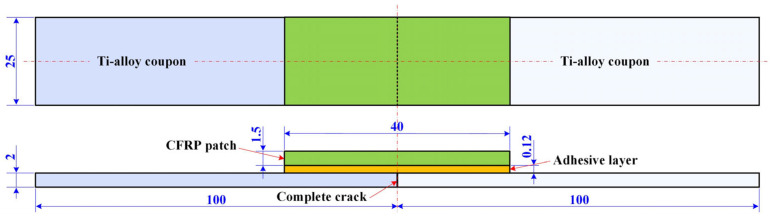
Geometrical configurations of repaired Ti-alloy specimens with single-sided composite patches (unit: mm).

A butt joint containing two Ti-alloy coupons and one single-sided composite patch bonded with adhesive film was employed as the test configuration to investigate the efficiency of three distinct repair methods applied to fully cracked metallic structures, as illustrated in Figure 1. Differing from the semi-cracked specimens in previous work [18,19], this configuration excluded the influence of residual stiffness and the strength of cracked metallic components on the repair performance so that only the effects of the repair methods were involved. The Ti-alloy coupon was made from Ti6Al4V with a geometry of 100 mm× 25 mm × 2 mm and provided by Shanghai Luhao Metal Co., Ltd., Shanghai, China. The adhesive film was SY-24C structural adhesive with a thickness of 0.12 mm and was provided by the Beijing Institute of Aerial Materials (Beijing, China). As for the patch, there were four different specifications, namely pre-cured unidirectional laminate, unidirectional (UD) prepreg, plain weave (PW) prepreg and wet-layup patches that corresponded to pre-curing, prepreg and wet-layup methods. For simplicity, the specimens repaired with pre-cure, UD prepreg, PW prepreg and wet-layup patches are represented by cases A, B, C and D, respectively. These patches were made from T300 carbon fibers and 7901 epoxy matrix resin provided by Shanghai Cedar Composites Technology Co., Ltd. (Shanghai, China). The main ingredient of the 7901 matrix was phenolic resin with a reactive flame retardant. The geometry of the patches was set to 40 mm × 25 mm × 1.5 mm. Each UD prepreg or dry fiber cloth thickness was about 0.15 mm, thus there were 10 plies in a composite patch and the total nominal thickness was 1.5 mm in cases A, B and D. As for the PW prepreg in case C, the single ply was 0.2 mm in thickness, with only 7 plies employed to obtain the total nominal thickness of 1.4 mm. The adhesive film and the composite prepreg were covered with anti-adhesive paper on both sides to prevent contamination prior to their use. The mechanical properties of the Ti-alloy coupon, film adhesive, cured UD and PW composite laminas are listed in Table 1.

### 2.2. Preparation of Repaired Specimens

The Ti-alloy coupons were cut with a water jet machine into the principal dimensions. The butt ends of the coupons were slightly ground to make flat and straight mating surfaces for the “crack.” The surface roughness of these coupons was R_a_ 24.1 μm measured with a 3D surface morphometer (Hirox MXB-5000REZ, Tokyo, Japan) within an evaluation length of 10 mm. In order to have a good bonding surface, the machined Ti-alloy coupons were first wiped with non-woven fabrics dipped with acetone to remove grease and dust preliminarily. Then they were cleaned with purified water in an ultrasonic cleaning machine for 10 min. The washed coupons were placed in an oven at 80 °C for 1 h to remove moisture. The well-prepared Ti-alloy coupons were wrapped with polytetrafluoroethylene films to avoid contamination before repair bonding.

The preparation of four distinct composite patch specifications from three repair methods was more complex than the treatment of Ti-alloy coupons. In the pre-cured method for case A, the UD laminate patches were cut from pre-cured [0]_10_ composite plate with a water jet machine. Then they were washed with anhydrous alcohol and dried in a drying oven ready to be used. For cases B and C in the prepreg method, the patch plies were cut from T300/7901 UD prepreg or PW prepreg before being attached to the cracked components. As for case D in the wet-layup method, the dry UD patch plies were first cut from fiber cloth and then immersed with on-site blended epoxy resin before use. During this process, the fiber cloth was prone to scattering. The saturation and volume content of the resin were of vital importance to the final quality of the wet-layup patches.

**Figure 2:**
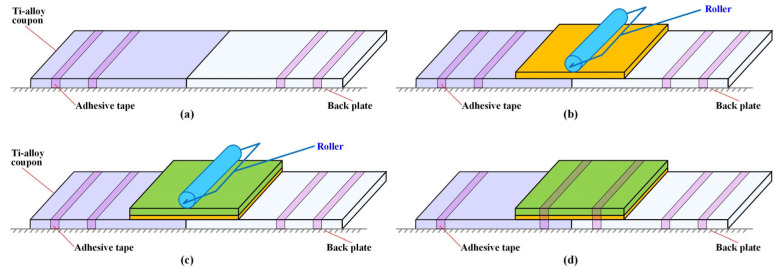
Preparation for cracked Ti-alloy specimen repaired with a composite patch. (**a**) Fixation of Ti-alloy coupons, (**b**) adhesive film layup, (**c**) composite patch layup, (**d**) fixation of well-prepared specimen.

The repair bonding process where the composite patch was applied to the cracked Ti-alloy component is presented in Figure 2. Firstly, two Ti-alloy coupons were fixed to a steel back plate with thin adhesive tape to form a butt joint (Figure 2a). Then, adhesive film was cut to the required size and pasted on to the butt zone after its bottom paper was uncovered. A roller was used to press the upper surface of the adhesive film to remove bubbles and gaps in order to make it firmly bond to the Ti-alloy coupons (Figure 2b). Thirdly, the prepared composite patch was overlaid on the adhesive film with a designed 0° fiber orientation after the papers on both mating surfaces were removed. Again, the roller was used to consolidate the patch, wet all the fibers, release air, and to conform to the coupons. The latter two steps were strictly repeated if more than one layer of prepreg was needed for repairing specimens in the cases of B, C and D in the prepreg and wet-layup methods (Figure 2c). Finally, the patch zone was bundled up with thin adhesive tape and fixed to the backing plate to avoid relative movement in the upcoming cure process (Figure 2d).

**Figure 3:**
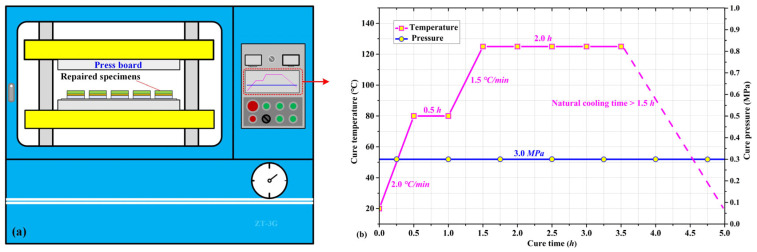
Cure process for cracked Ti-alloy specimens repaired with composite patches, (**a**) repaired specimens placed in automatic pressure precision machine, (**b**) curing temperature and pressure curves.

The four preformed repair specimen specifications were cured in an automatic pressure precision machine (ZG-3T, Dongguan, China) as shown in Figure 3a. The curing temperature and pressure curves are presented in Figure 3b. Since the SY-24C adhesive film and 7901 epoxy resin had the same curing temperature and duration, it was reasonable to choose these two materials for repair research. In the pressure machine, the preformed repair specimens together with the backing plates were placed between two parallel pressure boards with a predetermined cure pressure of 0.3 MPa applied to the specimens (Figure 3a). The specimens were first heated up from room temperature to 80 °C in 0.5 h, followed by a 0.5-h maintenance at this temperature for the initial cure. Then the cure temperature rose to 125 °C in 0.5 h and was kept at that temperature for 3 h for a thorough cure (Figure 3b). The specimens were naturally cooled through opening the air-tight door to release the residual stress caused during the curing process. The spilt prepreg and adhesive on both lateral sides of the repaired specimens were cut out to make their final dimensions. It should be noted that although the UD patches in cases A, B and D were 0.1 mm thicker than the PW patches in cases C, the former were easily flattened due to them lacking lateral constraints to the longitudinal fiber bundles. Fiber bundles and epoxy resin spilt out easily under the cure pressure, resulting in a reduced thickness than the nominal value. However, it was not the case for the PW patch since the fiber bundles of warp yarn and weft yarn constrained mutually, making them hard to spill out. Thus, the final thickness difference between UD and PW patches was much smaller than 0.1 mm and its influence on repair efficiency and failure mode could be ignored.

### 2.3. Mechanical Test for Repaired Specimens

**Figure 4:**
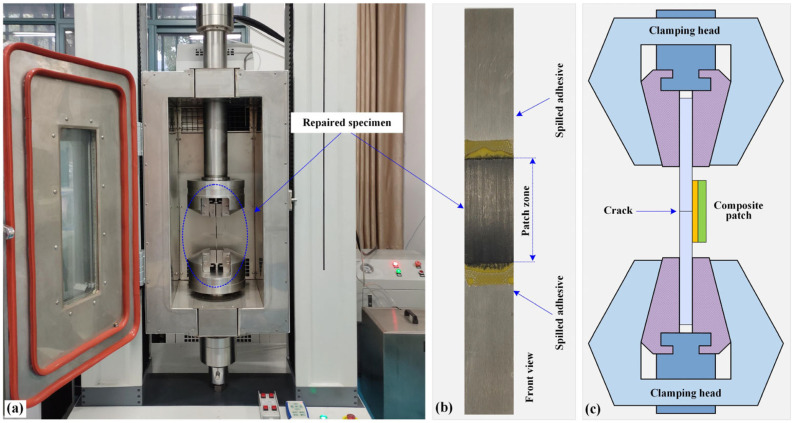
Test setups for repaired specimens: (**a**) testing machine with clamped specimen, (**b**) front view of repaired specimen, (**c**) hydraulic gripping fixture with clamped specimen.

The specimens repaired with different methods were statically loaded to evaluate their repair effectiveness. The quasi-static tensile tests were conducted in a universal testing machine (Pootae 100GDW-60, Dongguan, China) with a load capability of 100 kN, as shown in Figure 3a. The relative error of force-displacement measurement and control accuracy was within ±1%. Before testing, two ends of the repaired specimen were held with hydraulic clamping fixtures at a distance of 30 mm as shown in Figure 4a. Then, all the tests were performed with a constant displacement rate of 1.0 mm/min during which the data of load–displacement curves were continuously recorded. Loading stopped when the repaired specimens ruptured and the load-displacement curves drastically dropped. Each specimen specification was repeated at least five times. After testing, the damage morphology and failure mode of the broken specimens were elaborately observed and analyzed. It is worth mentioning that for each patch specification, the five specimens were made in two batches (see Section 2.2): three in the first and two in the second to evaluate the stability of repair process.

## 3. Results and Discussion

### 3.1. Loading Behaviors of Repaired Structures

**Figure 5:**
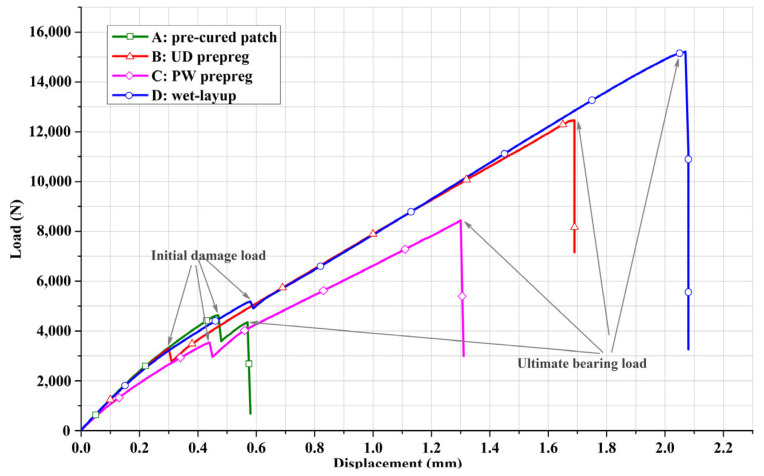
Typical load-displacement curves of four repaired specimen specifications.

The typical load-displacement curves of the four specimen specifications from the three repair methods are presented in Figure 5. Generally, all the curves went through three phases: elastic-linear, damage propagation and complete fracture regardless of their final bearing capacity. For each load-displacement curve, these three phases were divided into two key points: the initial damage load point and the ultimate bearing load point. Commonly, the value of the former was smaller than that of the latter since the repaired structure continued to bear load increments after damage initiated and propagated in the repaired zone. There is, however, an exception to the pre-cured method or case A, where the phase of damage propagation was very short and the ultimate peak value was visibly smaller than that at the damage initiation point. This unique phenomenon indicated that the failure mechanism of the pre-cured method was different from the prepreg and wet-layup methods since it was adhesively bonded, while the other two were essentially co-cure joined. It should also be noted that the curve slope and ultimate bearing load of the PW prepreg patch in case C were much smaller than those of the UD prepreg patch in case B although they were prepared with the same carbon fibers and epoxy resin; this gap cannot be ascribed to the small thickness difference but to the difference of the 0° fiber content in the loading direction.

**Figure 6:**
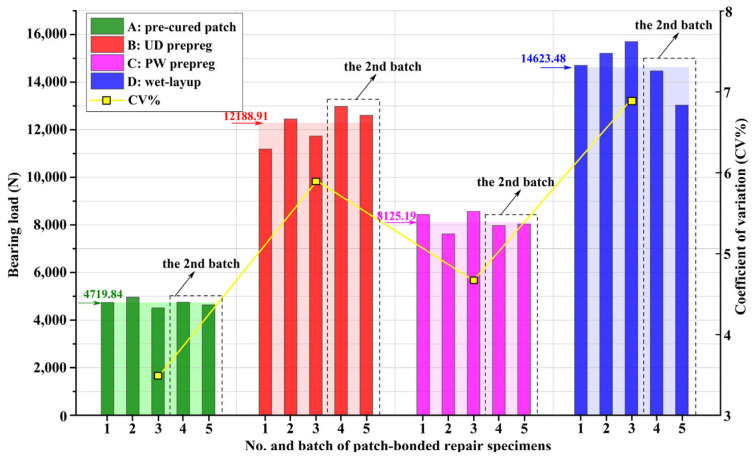
The ultimate bearing loads and the corresponding coefficients of variation (CV) of all repaired specimens from two separate preparation batches.

In order to evaluate the processing stability of the three repair methods, five repaired specimens in each patch specification were fabricated in two separate batches as mentioned earlier. The ultimate bearing loads of all the repaired specimens and their corresponding coefficients of variation (CV) are presented in Figure 6. It can be observed that for the specimens with the pre-cured and the WP patches in cases A and C, respectively, the ultimate bearing loads of the second batch were roughly the same as those in the first one. The CV of case A was only 3.51%, which was smaller than that of case C, which was about 4.67%. For the UD prepreg patch in case B, the ultimate bearing loads of the second batch were visibly greater than those of the first batch and because of this, the CV was larger than the PW patch in case C, at about 5.91%. As for the wet-layup patch in case D, the ultimate bearing loads of the second batch were obviously smaller than the first, thus the CV was the greatest of all at about 6.89%. Therefore, it can be concluded that the process stability of the pre-cured method was the best, followed by the PW and UD prepreg patches in the prepreg method. The wet-layup method presented the most unstable repair process because applying the matrix resin to unidirectional dry fiber cloth to form wet and sticky patch plies at the repair site was hard to standardize, and depended heavily on personal experience.

**Figure 7:**
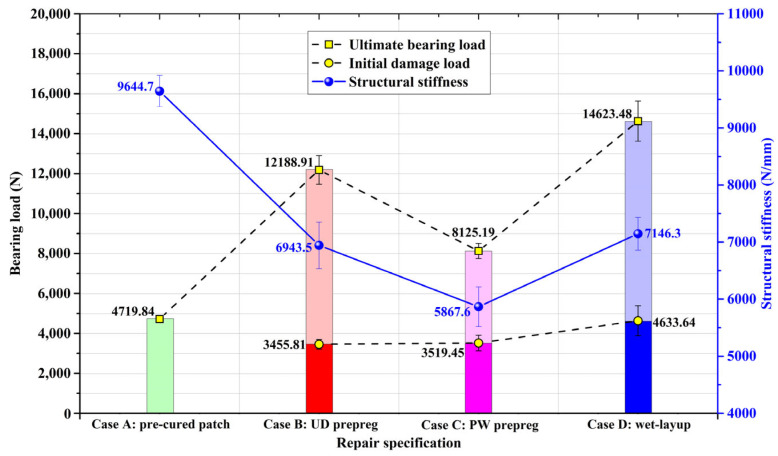
Structural stiffness, initial damage, and ultimate bearing loads of repaired specimens from four patch specifications.

The average structural stiffness, initial damage, and the ultimate bearing loads of four repaired specifications abstracted from their load-displacement curves are presented in Figure 7 to evaluate the repair effectiveness. Although the pre-cured patch in case A, UD prepreg patch in case B and wet-layup patch in case D have the same amount of 0° plies in loading direction, the repaired specimens in case A possessed the highest structural stiffness among them, about 9644.7 N/mm, followed by cases D and B in sequence, about 7146.3 N/mm and 6943.5 N/mm, respectively, which evidences that the repair method could partly determine the final stiffness of repaired structures. Since the 0° fiber content of PW prepreg patch in loading direction in case C was half of that in cases A, B and D, its stiffness was only 5867.6 N/mm, demonstrating that the architecture of composite patch could also affect the structure stiffness. In terms of loading behavior, the repaired specimens in case D possessed the highest ultimate bearing load of 14,623.48 N, closely followed by the repaired specifications of case B and then case C, about 12,268.91 N and 8125.19 N, separately. The ultimate bearing load in case A was only 4179.84 N owing to the bad bonding interface. As for initial damage load, it was 4633.64 N in case D, which was nearly equivalent to the ultimate bearing load of case A. In contrast, the initial damage load of prepreg method was much smaller, about 3455.81 N and 3519.45 N for case B and C, individually. It can be concluded that the on-site prepared wet-layup patch can achieve better interfacial binding force than the readymade prepreg patches because of the rich matrix resin. However, the sufficient resin applied to the fiber cloth could not distribute evenly and fill out the space between all of the fibers, which conversely pushes up the CV in patch preparation process.

### 3.2. Interfacial Bonding Microstructures

**Figure 8:**
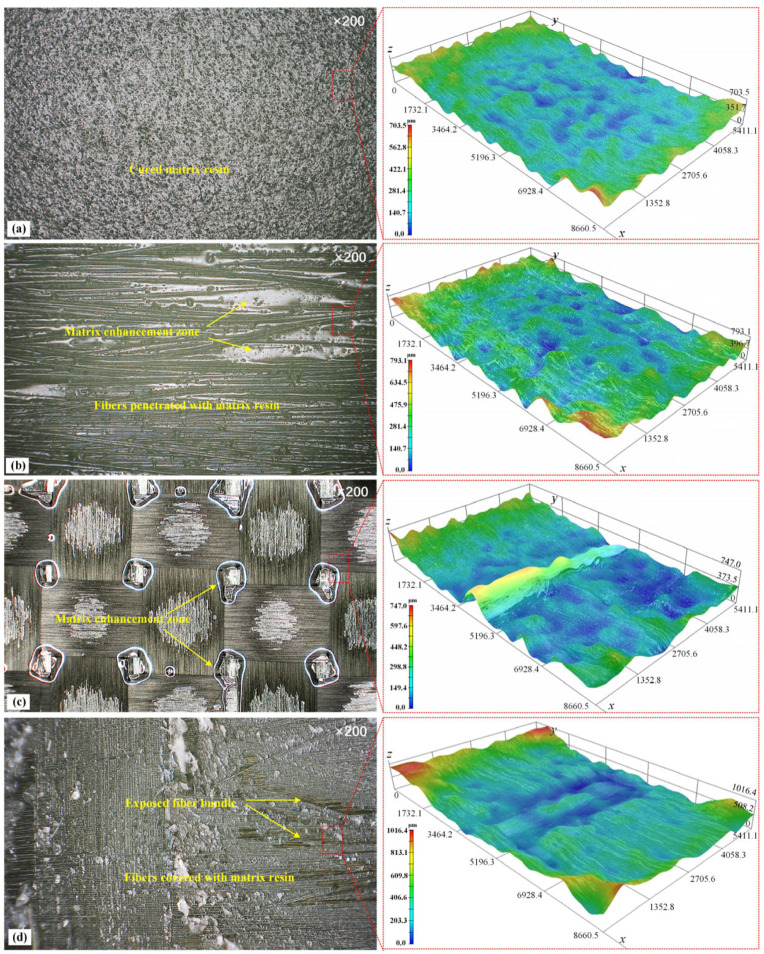
Surface morphology of composite patches made via different methods: (**a**) pre-curing method, (**b**) unidirectional prepreg patch, (**c**) weave prepreg patch, (**d**) wet-layup method.

The surface morphologies of the composite patches made from four repair specifications were observed with an automatically zoomed 3D surface measurement instrument (NT1100, Veeco Wyko, Tucson, AZ, USA) to figure out the mechanism of interfacial bonding behaviors in different cases (Figure 8). In the pre-cured patch, the fibers were covered with cured epoxy resin which formed a light-reflecting and low-energy surface. The surface roughness was within 351.7 μm, which was too smooth to be infiltrated with a structural adhesive to generate a mechanical engagement force on the bonding interface (Figure 8a). In the UD prepreg patch, the fiber bundles together with the striped matrix resin enrichment zones were easily observed. The undulated side-faces of the fibers and the uncured matrix resin formed a relatively coarse and sticky surface for repair bonding, which greatly improved the interface binding force (Figure 8b). The PW prepreg patch was similar to the UD patch. The difference was that the intertwined warp and weft fiber bundles formed a squared and uneven surface with a roughness of 597.6 μm. The resin enrichment zones were coincidentally at the intersection points of the warp and weft yarns, whereas the resin deficiency zones were in the center of the squares (Figure 8c). In the wet-layup patch, the fiber bundles were bound loosely with a thermal fuse to form waved, unidirectional fiber cloth in which sufficient gaps or space were reserved for the penetration of the applied matrix resin. The prepared wet-layup patches with the flexible fiber bundles and abundant epoxy resin fitted the Ti-alloy coupons seamlessly, making a well-bonded interface to restore the structural load transfer path (Figure 8d).

**Figure 9:**
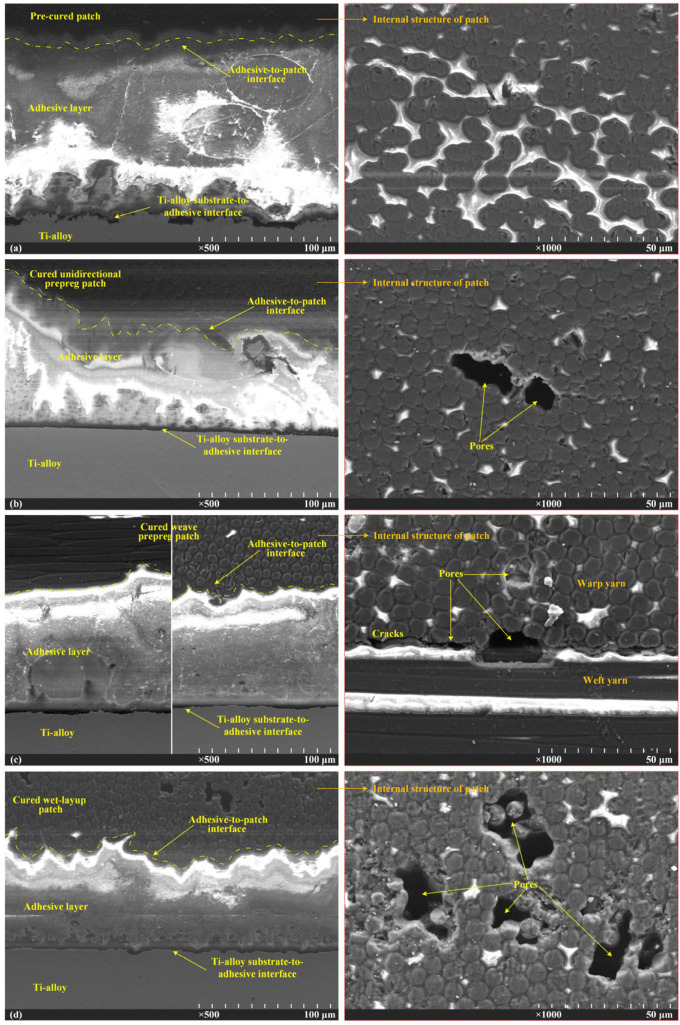
The microstructures of the bonding interfaces between Ti-alloy coupons and composite patches: (**a**) pre-curing method, (**b**) unidirectional prepreg patch, (**c**) weave prepreg patch, (**d**) wet-layup method.

The microstructures of the bonding interfaces between the Ti-alloy and the four composite patch specifications were inspected via a scanning electron microscope (JEOL SEM5600LV, Tokyo, Japan), as seen in Figure 9. All of the bonding interfaces contained five layers: the Ti-alloy substrate, the Ti-alloy-to-adhesive interface, the adhesive layer, the adhesive-to-patch interface, and the patch substrate; their thickness and micromorphology varied with the distinct repair methods. For the pre-cured patch interface in case A, the adhesive layer was wide because it could only be squeezed out of the bonding area via the pre-cured laminate patch instead of penetrating into it; there was a clear demarcation interface between the adhesive layer and the composite patch. A high level of peeling stress was induced via the eccentric load patch in the wide adhesive layer and this resulted in cohesive failure [13]. The only advantage of the pre-cured method was that the composite patch was compact inside and no pore was observed (Figure 9a). For both the UD and the PW prepreg patches in cases B and C, the width of the adhesive layer was much narrower than that in case A. There was no apparent boundary between the adhesive layer and the prepreg patch. Instead, they integrated, penetrated, and even interlocked with each other, forming a well-bonded interface. However, the disadvantage was that the pores were observed in the handmade patches, indicating that the air was not completely discharged during the rolling and curing, undermining the strength of the patches (Figure 9b,c). It should be noted that in case C, the pores often appeared near the intersection zone of the warp and weft fiber bundles of the PW prepreg patch and became the crack cores which developed into observable cracks when subjected to the external loading (Figure 9c). As for the wet-layup patch in case D, the fusion between the adhesive layer and the carbon fiber cloth immersed with epoxy resin was strengthened when compared with cases B and C. Thus, the interface between them presented highly jigsaw-like patterns which were regarded as good bonding interfaces. However, the pores were more prone to formation in the wet-layup patch since it was hard to ensure the uniformity of the manually immersed matrix resin in the dry carbon fiber cloth (Figure 9d). The repair methods in cases B, C and D using the co-curing process with soft uncured patches conformed more easily to the complex geometries of the cracked structures than the rigid pre-cured patch in case A. Comparatively, the prepreg method was a compromised choice that prevailed against the others in terms of the quality of the bonding interface, the patch compactness, and the compaction process that needs to be improved to reduce patch porosity.

### 3.3. Damage and Failure Modes

**Figure 10:**
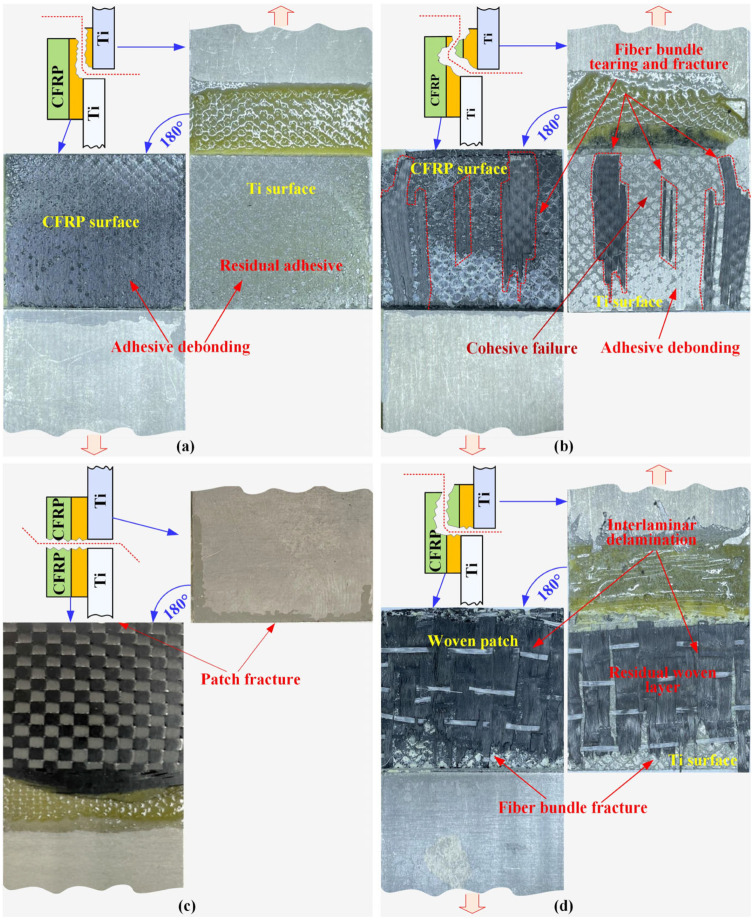
Failure morphology of repaired specimens with different repair methods: (**a**) pre-curing method, (**b**) unidirectional prepreg patch, (**c**) weave prepreg patch, (**d**) wet-layup method.

On the cracked Ti-alloy panel repaired with the composite patch, the external load was transmitted along the path from the Ti-alloy substrate, the Ti-alloy-to-adhesive interface, the adhesive layer, the adhesive-to-patch interface to the composite patch sequentially, during which, the weakest part in such a repair system determined the load carrying capacity of the repaired specimen. This weakest part was identified through observation of the failure modes in the broken repaired structures. The failure morphologies of the four repaired specifications are presented in Figure 10.

In case A, the pre-cured patch was entirely peeled off from the upper Ti-alloy coupon and attached to the lower one. The residual adhesive film mainly adhered to the upper Ti-alloy surface, while the mating surface of the composite patch was light-reflecting. The fractured mating surfaces exhibited an adhesive failure mode in the adhesive-to-patch interface, demonstrating that the pre-cured composite patch was not suitable for metallic repair (Figure 10a). In case B, the UD prepreg patch detached from the upper Ti-alloy coupon and presented three mixed failure modes. The clean and smooth zones on the surface of the upper Ti-alloy was adhesive or debonding failure on the Ti-alloy-to-patch interface which occupied about 20% of the fracture surface. Some fragments of the bottom ply were torn out from the UD prepreg patch and were tightly attached to the Ti-alloy surface, presenting an adherend failure mode. The other 50% of the surface was a cohesive failure since the residual adhesive film was observed on the two uneven mating surfaces. It was a transitional stage where the failure mode transitioned from the adhesive failure to the prepreg ply failure, indicating that these vital layers had similar strength in the repair system (Figure 10b). In case D, the main failure mode was the interlaminar delamination of the wet-layup patch, accompanied by the fracture of the fiber bundles and the cohesive failure at the end of the upper Ti-alloy coupon; the adhesive strength was therefore much greater than the interlaminar strength of the wet-layup patch. Thus, the patch itself was the weakest part of the repair system and its interlayer performance must be purposefully enhanced (Figure 10d). The only distinctive failure mode occurred in case C, where the PW weave patch broke in half along the central crack. This failure of morphology was reasonable when considering that the 0° fiber content in the PW prepreg patch in the loading direction was only half of that of the UD patch. It is believed that the PU prepreg is not an ideal alternative to UD patches with a diverse stacking sequence when the same grade carbon fiber prepreg is employed for structural reparation. (Figure 10c).

## 4. Conclusions

The aim of this research was to seek a manual composite patch repair method to apply to cracked metallic structures. For this purpose, Ti-alloy butt joints bonded with pre-cured, UD prepreg, PW prepreg and wet-layup composite patches were employed for four repair specifications. The repair efficiency and processing stability were evaluated via the load carrying capacity, the microstructure of the bonding interface and the structural failure morphology. The findings and conclusions are summarized as follows:

(1) In terms of loading performance, the wet-layup method provided the best initial damage and ultimate load bearing with good structural stiffness. This was closely followed by the UD prepreg patch. The pre-cured method attained the highest structural stiffness, yet the worst load-carrying capacity owing to the premature debonding on the adhesive-to-patch interface. The WP prepreg patch presented poor stiffness and strength features because of the low 0° fiber content in the loading direction.
(2) As for the repair process, although the pre-cured method produced dense composite patches and implemented easily with the minimum CV, the poor interfacial binding ability blocked its application. Conversely, the wet-layup method presented a highly jigsaw-like mating interface, yet also a porous patch with the maximum CV. Comparatively, the prepreg method, especially the UD prepreg patch, achieved both a good bonding interface and a less porous composite patch with a relatively low CV in loading capacity.
(3) The fail morphology identified the weakest part of the repaired structures. The patch delamination in the wet-layup method suggests that the interlaminar strength of the patch is much weaker than that of the adhesive film. In the prepreg method, the PW patch broke in half since it did not have sufficient strength in the loading direction, while in the UD patch, the failure transitioned from adhesive to ply failure, indicating that the patch and the adhesive layer reached a balance in strength.

Conclusively, when balancing the repair efficiency and the processing stability, the use of the UD prepreg patch was a compromised or suboptimal method for repairing cracked metallic structures. The PW prepreg patch was still promising when a much higher grade of carbon fiber was employed.

## Figures and Tables

**Table 1:** Mechanical properties of Ti-alloy coupon, adhesive film and cured UD and PW composite prepreg laminas.

Ti6Al4V Alloy		Adhesive SY-24C		UD Lamina T300/7901		Weave Lamina T300/7901	
*E* (GPa)	112	*E* (MPa)	5750	*E*_1_ (GPa)	125	*E*_1_, *E*_2_ (GPa)	50
*G* (GPa)	43	*G* (MPa)	1920	*E*_2_, *E*_3_ (GPa)	11.3	*E*_3_ (GPa)	3.5
*ν*	0.32	*σ* (MPa)	451.6	*ν*_12_, *ν*_13_	0.3	*ν*_12_	0.25
*σ*_b_ (MPa)	930	*τ* (MPa)	225.8	*ν*_23_	0.42	*ν*_13_, *ν*_23_	0.48
*σ*_s_ (MPa)	860	$G_{n}^{C}$ (Nmm^−1^)	0.48	*G*_12_, *G*_13_ (GPa)	5.43	*G*_12_ (GPa)	2.98
Δ (%)	10	$G_{s}^{C}$, $G_{t}^{C}$ (Nmm^−1^)	0.64	*G*_23_ (GPa)	3.98	*G*_13_, *G*_23_ (GPa)	2.31

## Data Availability

The data presented in this study are available on request from the corresponding author.
